# Supplementary material for: The linear ubiquitin chain assembly complex regulates TRAIL‐induced gene activation and cell death
Source: EMBO J. 2017 Mar 3;36(9):1147–66. doi: 10.15252/embj.201695699 (PMC5412822; doi:10.15252/embj.201695699)

Appendix - Table of contents

- Legends for Appendix Figures S1, 2 and 3
- Appendix Figures S1, 2 and 3

Appendix Figure S1 (related to Figure 1) - HOIP limits TRAIL-induced cell death

A, B, E, F Cell lysates from the indicated cell lines were analysed by Western Blot.

C Control and HOIP KO HeLa cells were stimulated for 24 hours with iz-TRAIL at the indicated concentrations. Cell viability was determined (n=4; mean+/-SEM). *p < 0.05; statistics were performed using Mann-Whitney U-test.

D Control and HOIP KO HeLa cells, pre-treated for 1 hour with zVAD as indicated, were stimulated with iz-TRAIL (100 ng/mL) for 24 hours before viability was evaluated (n=3; mean+/-SEM). *p<0.05; statistics were performed using Mann-Whitney U-test.

Appendix Figure S2 (related to Figure 3) - HOIP is cleaved by caspase-8 upon TRAIL and TNF stimulation

A HOIP-TAP was immunoprecipitated using anti-FLAG beads. Samples were incubated for 2 hours with the indicated active caspases and analysed by Western blot. The black arrowhead indicates the cleaved form of HOIP.

B MCF-7 WT cells were transfected with the indicated combinations of siRNA control, targeting caspase-8, caspase-6 or caspase-10. 72 hours later, cells were pre-treated for 1 hour with cycloheximide (0.5 µg/mL) and further treated with His-TNF (200 ng/mL) for the indicated times. Lysates were analysed by Western blot. The black arrowhead indicates the cleaved form of HOIP.

C HeLa WT cells were transfected with the indicated combinations of siRNA control, targeting caspase-8 or caspase-3. 72 hours later, cells were pre-treated for 1 hour with cycloheximide (0.5 µg/mL) and further treated with His-TNF (200 ng/mL) for the indicated times. Lysates were analysed by Western Blot. Black arrowheads indicate the cleaved forms of HOIP.

D HT29 WT cells were treated with iz-TRAIL (1 µg/mL) for the indicated times. Lysates were analysed by Western Blot. Black arrowhead indicates the cleaved form of HOIP.

E TNF KO MEFs were pre-treated for 1 hour with cycloheximide (0.5 µg/mL) and the TAK1 inhibitor (5Z)-7-Oxozeaenol (1µM), as indicated. Cells were further treated with His-TNF (200 ng/mL) for the indicated times. Lysates were analysed by Western Blot. The black arrowhead indicates the cleaved form of HOIP.

Appendix Figure S3 (related to Figure 7) – IKK and MAPK pathways contribute to TRAIL- and TNF-induced cytokine production

A HeLa WT, pre-treated for 1 hour with TPCA-1 (10 µM), SP600125 (15 µM), Losmapimod (5 µM) or PD184352 (1 µM) as indicated, were treated with iz-TRAIL (200 ng/mL) for the indicated times. Lysates were analysed by Western blot.

B HeLa WT, pre-treated for 1 hour with QVD (10 µM) and TPCA-1 (10 µM), SP600125 (15 µM), Losmapimod (5 µM) or PD184352 (1 µM) as indicated, were treated with iz-TRAIL (100

ng/mL) or His-TNF (50 ng/mL) for 24 hours as indicated. IL-8 and CCL-2 concentrations in cell supernatants were measured via ELISA (n=4; mean +/- SEM). Lysates were analysed by Western blot.

C A549 WT, pre-treated for 1 hour with QVD (10 µM) and TPCA-1 (10 µM), SP600125 (15 µM), Losmapimod (5 µM) or PD184352 (1 µM) as indicated, were treated with iz-TRAIL (100 ng/mL) or His-TNF (50 ng/mL) for 24 hours as indicated. IL-8 and CCL-2 concentrations in cell supernatants were measured via ELISA (n=4; mean +/- SEM). Lysates were analysed by Western blot.

Appendix Figure S1

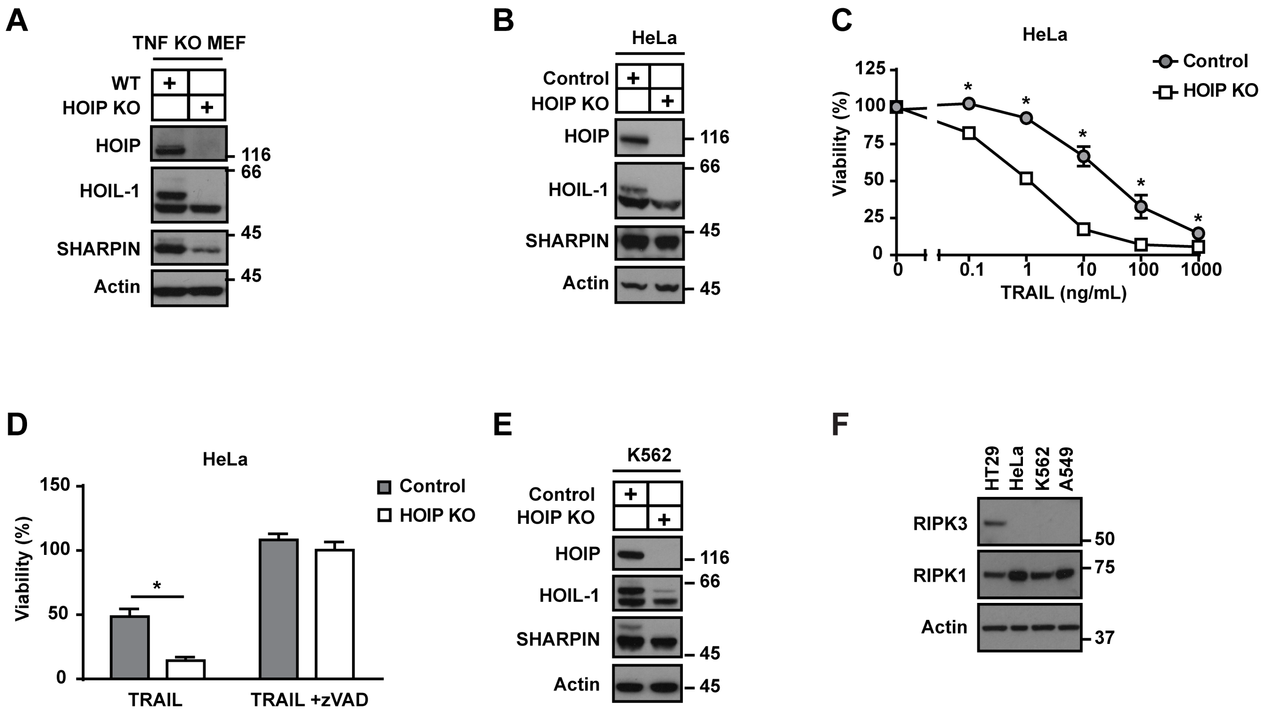

Appendix Figure S2

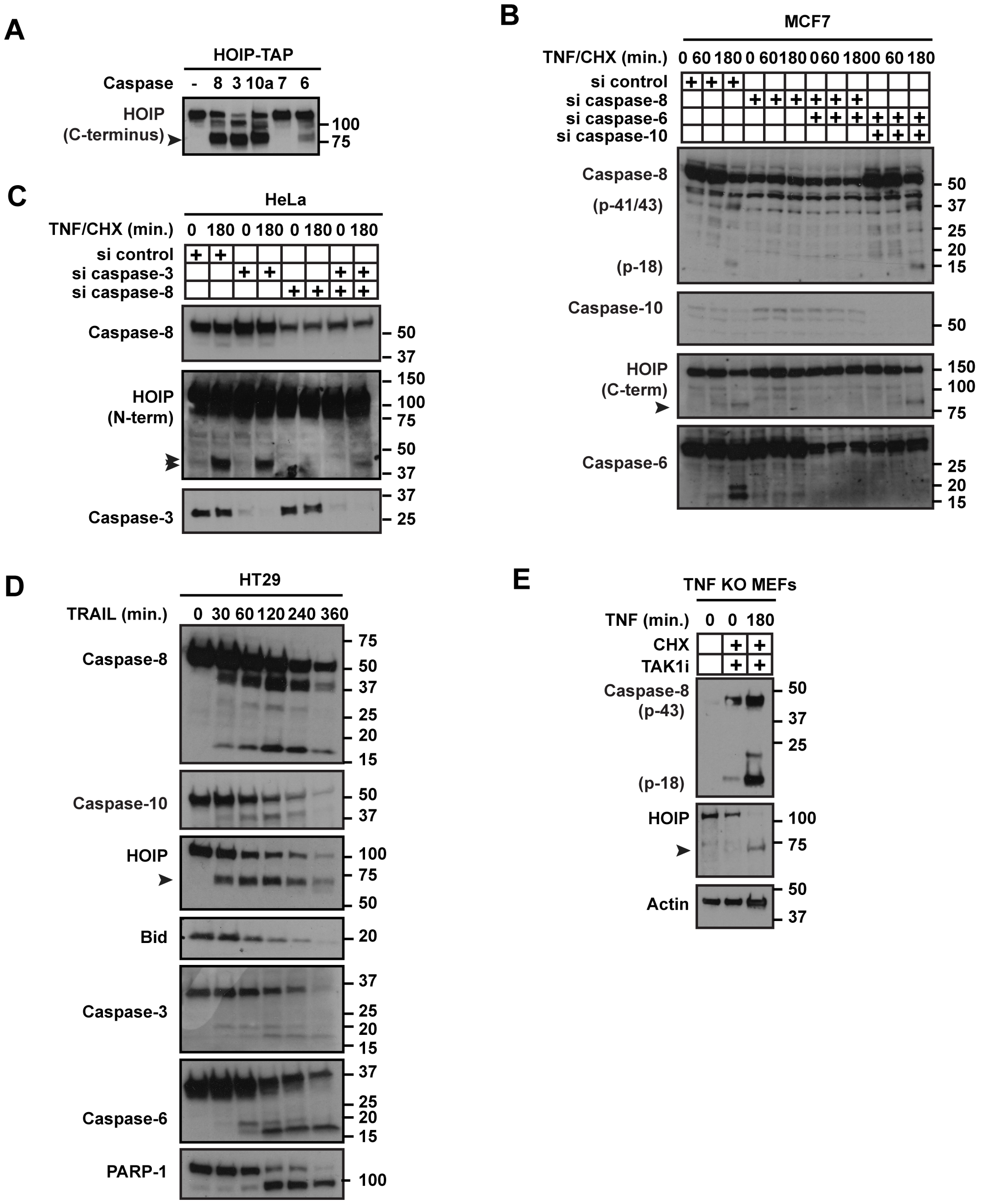

Appendix Figure S3

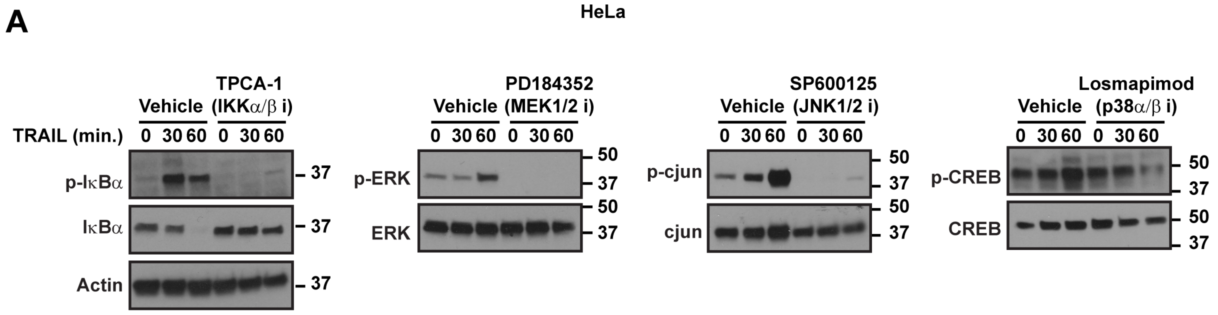

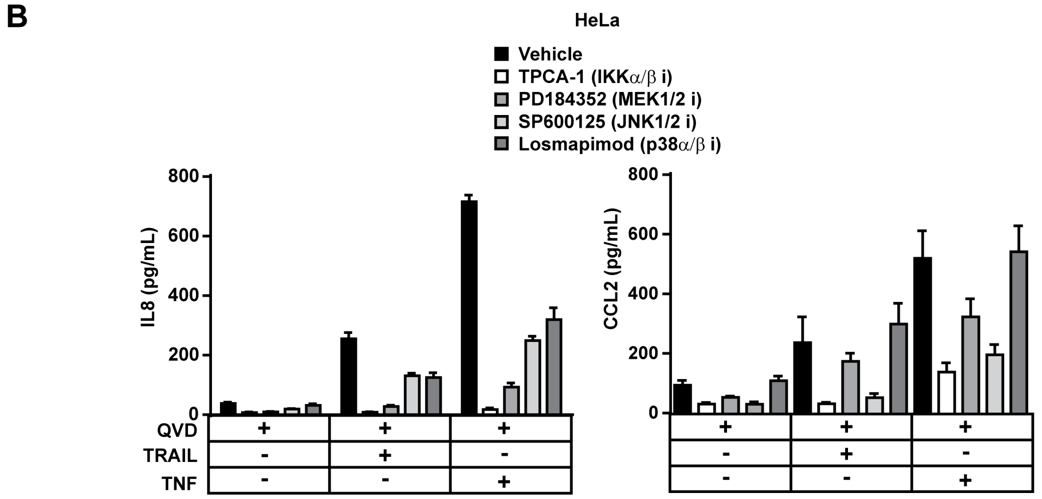

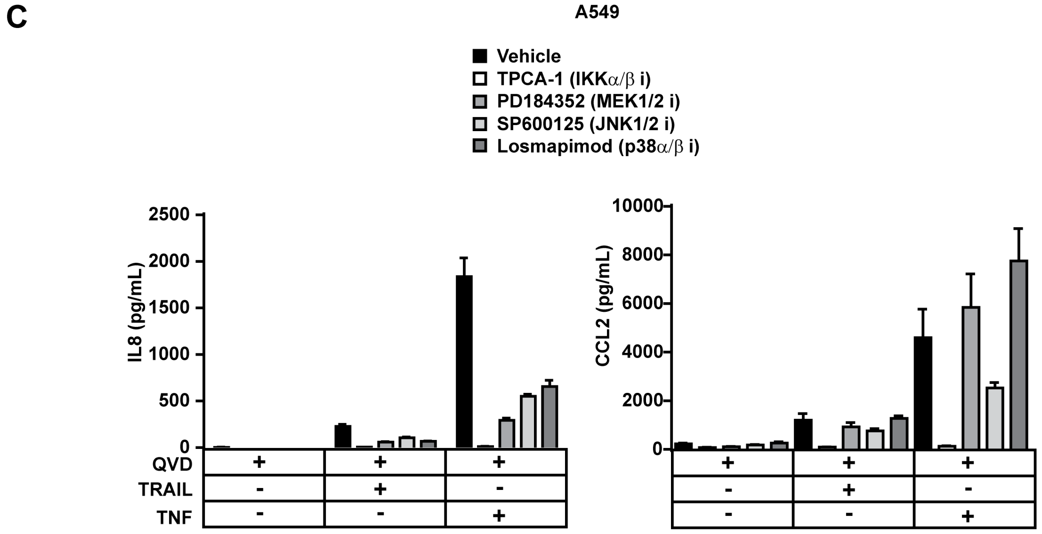

Supplement: Supplementary file 1 — Appendix [file EMBJ-36-1147-s001.pdf]
