# Supplementary material for: The linear ubiquitin chain assembly complex regulates TRAIL‐induced gene activation and cell death
Source: EMBO J. 2017 Mar 3;36(9):1147–66. doi: 10.15252/embj.201695699 (PMC5412822; doi:10.15252/embj.201695699)
Supplement: Supplementary file 2 — Expanded View Figures PDF [file EMBJ-36-1147-s002.pdf]

Expanded View Figures

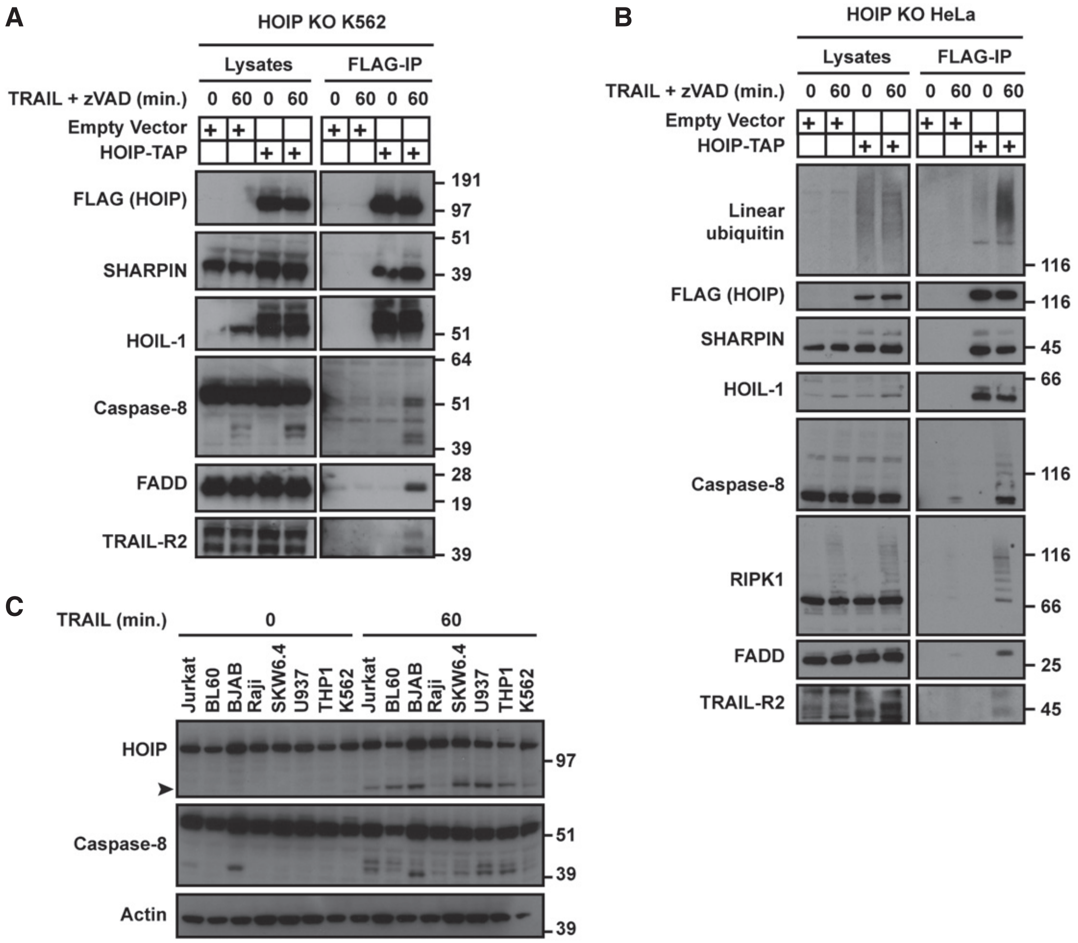

Figure EV1. LUBAC is recruited to and cleaved in TRAIL complex I (related to Fig 2).

A HOIP KO K562 cells reconstituted with empty vector or HOIP-TAP, pre-treated with zVAD for 1 h, were stimulated with iz-TRAIL (1 μg/ml) for 1 h as indicated. HOIP-TAP was immunoprecipitated using anti-FLAG beads, and samples were analysed by Western blot.

B HOIP KO HeLa cells reconstituted with empty vector or HOIP-TAP, pre-treated with zVAD for 1 h, were stimulated with iz-TRAIL (500 ng/ml) for 1 h as indicated. HOIP-TAP was immunoprecipitated using anti-FLAG beads, and samples were analysed by Western blot.

C The indicated cell lines were treated with or without iz-TRAIL (1 μg/ml) for 1 h and lysates were analysed by Western blot. The black arrowhead indicates the cleaved form of HOIP.

© 2017 The Authors

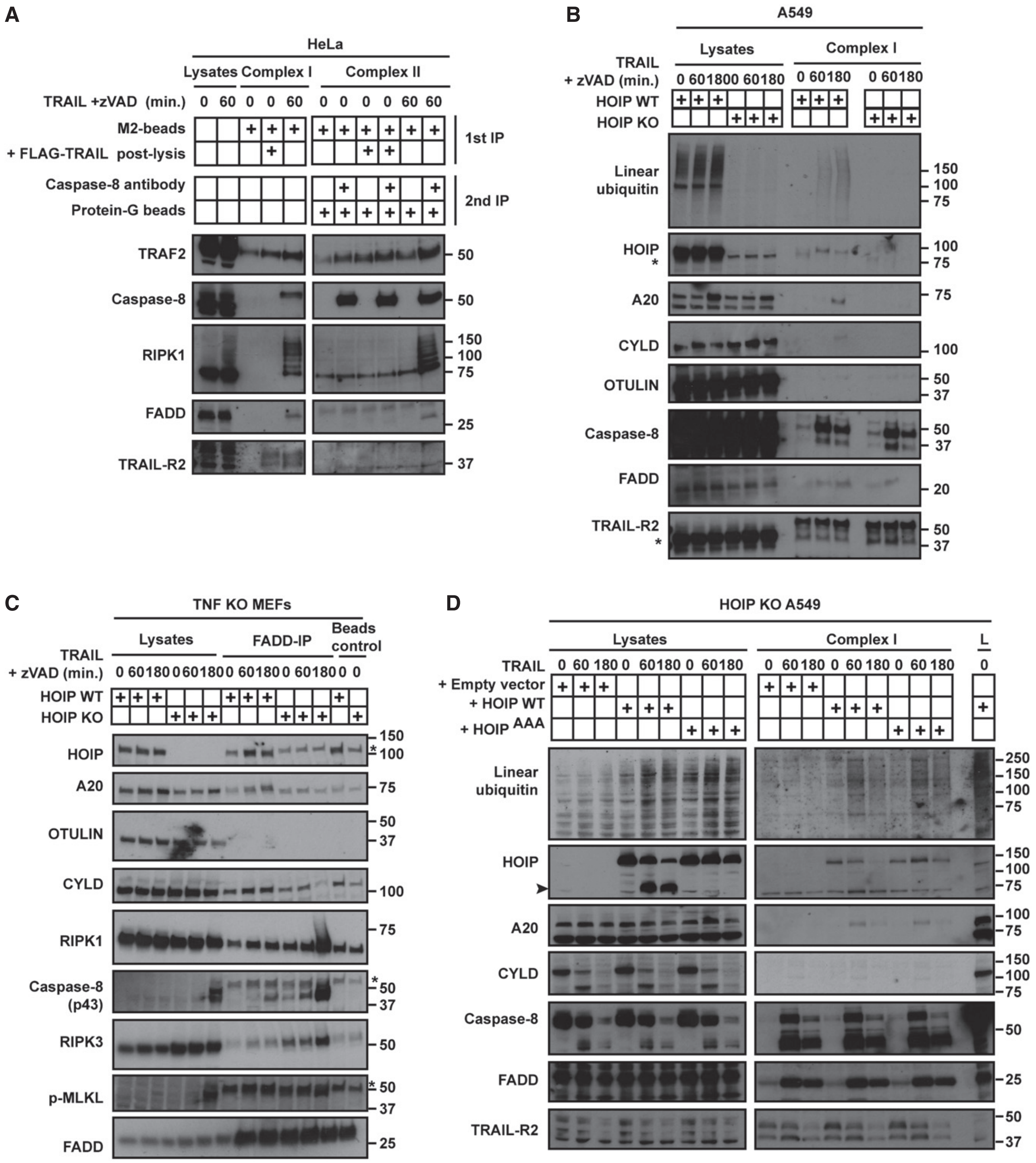
A
HeLa
Lysates Complex I Complex II
TRAIL +zVAD (min.)
M2-beads
+ FLAG-TRAIL post-lysis
1st IP
Caspase-8 antibody
Protein-G beads
2nd IP
TRAF2
Caspase-8
RIPK1
FADD
TRAIL-R2
B
A549
Lysates Complex I
TRAIL + zVAD (min.)
HOIP WT
HOIP KO
Linear ubiquitin
HOIP
A20
CYLD
OTULIN
Caspase-8
FADD
TRAIL-R2
C
TNF KO MEFs
Lysates FADD-IP Beads control
TRAIL + zVAD (min.)
HOIP WT
HOIP KO
HOIP
A20
OTULIN
CYLD
RIPK1
Caspase-8 (p43)
RIPK3
p-MLKL
FADD
D
HOIP KO A549
Lysates Complex I L
TRAIL
+ Empty vector
+ HOIP WT
+ HOIP AAA
Linear ubiquitin
HOIP
A20
CYLD
Caspase-8
FADD
TRAIL-R2

Figure EV2.

Figure EV2. A20 and CYLD are recruited to TRAIL complex I and II in a HOIP-dependent manner (related to Fig 4).

A HeLa cells pre-treated with zVAD for 1 h were stimulated with FLAG-lz-TRAIL (500 ng/ml) for 1 h as indicated. The TRAIL complex I was immunoprecipitated via anti-FLAG beads. Complex II was isolated by immunoprecipitating caspase-8 from complex I-depleted lysates. Additional controls are provided as indicated for each immunoprecipitation. Western blot were performed using the indicated antibodies.

B Control and HOIP KO A549 cells, pre-treated for 1 h with zVAD, were treated with FLAG-lz-TRAIL (500 ng/ml) for the indicated times. The TRAIL complex I was immunoprecipitated via anti-FLAG beads. Western blot was performed using the indicated antibodies. * indicates unspecific bands.

C WT and HOIP-deficient TNF KO MEFs, pre-treated for 1 h with zVAD, were stimulated with iz-TRAIL (1 μg/ml) for the indicated times. FADD-containing complexes were immunoprecipitated and analysed by Western blot. * indicates unspecific bands.

D HOIP KO A549 cells reconstituted with empty vector, HOIP WT or $HOIP^{AAA}$ were treated with FLAG-lz-TRAIL (500 ng/ml) for the indicated times. TRAIL complex I was immunoprecipitated via anti-FLAG beads. Western blot was performed using the indicated antibodies. L: lysate. The black arrowhead indicates the cleaved form of HOIP.

Figure EV3. HOIP promotes TRAIL- and TNF-induced gene-activatory signalling (related to Fig 7).

A WT, control and two different clones of HOIP KO K562 cells were stimulated for 1 h with iz-TRAIL at the indicated concentrations. Lysates were analysed by Western blot.

B BMDMs with 4-OHT-inducible deletion of HOIP from $CreERT2^{+}$ *$Hoip^{fl/fl}$* mice were stimulated with iz-TRAIL (100 ng/ml) for the indicated times. Lysates were analysed by Western blot.

C Control and HOIP KO HeLa cells were stimulated for the indicated times with His-TNF (100 ng/ml), and lysates were analysed by Western blot.

D BMDMs with 4-OHT-inducible deletion of HOIP from $CreERT2^{+}$ *$Hoip^{fl/fl}$* mice were stimulated with His-TNF (50 ng/ml) for the indicated times. Lysates were analysed by Western blot.

E K562 WT cells, pre-treated with TPCA-1 for 1 h as indicated, were treated with iz-TRAIL (1 μg/ml) for the indicated times. Lysates were analysed by Western blot.

F Control (CTRL) and HOIP KO HeLa cells, pre-treated with cycloheximide (CHX) for 1 h as indicated, were treated with the indicated concentrations of iz-TRAIL for 24 h before viability was determined (n = 3; mean ± SEM). Significant differences between corresponding HOIP KO and control groups are shown as follows: $*P < 0.05$; statistics were performed using Mann–Whitney *U*-test.

© 2017 The Authors

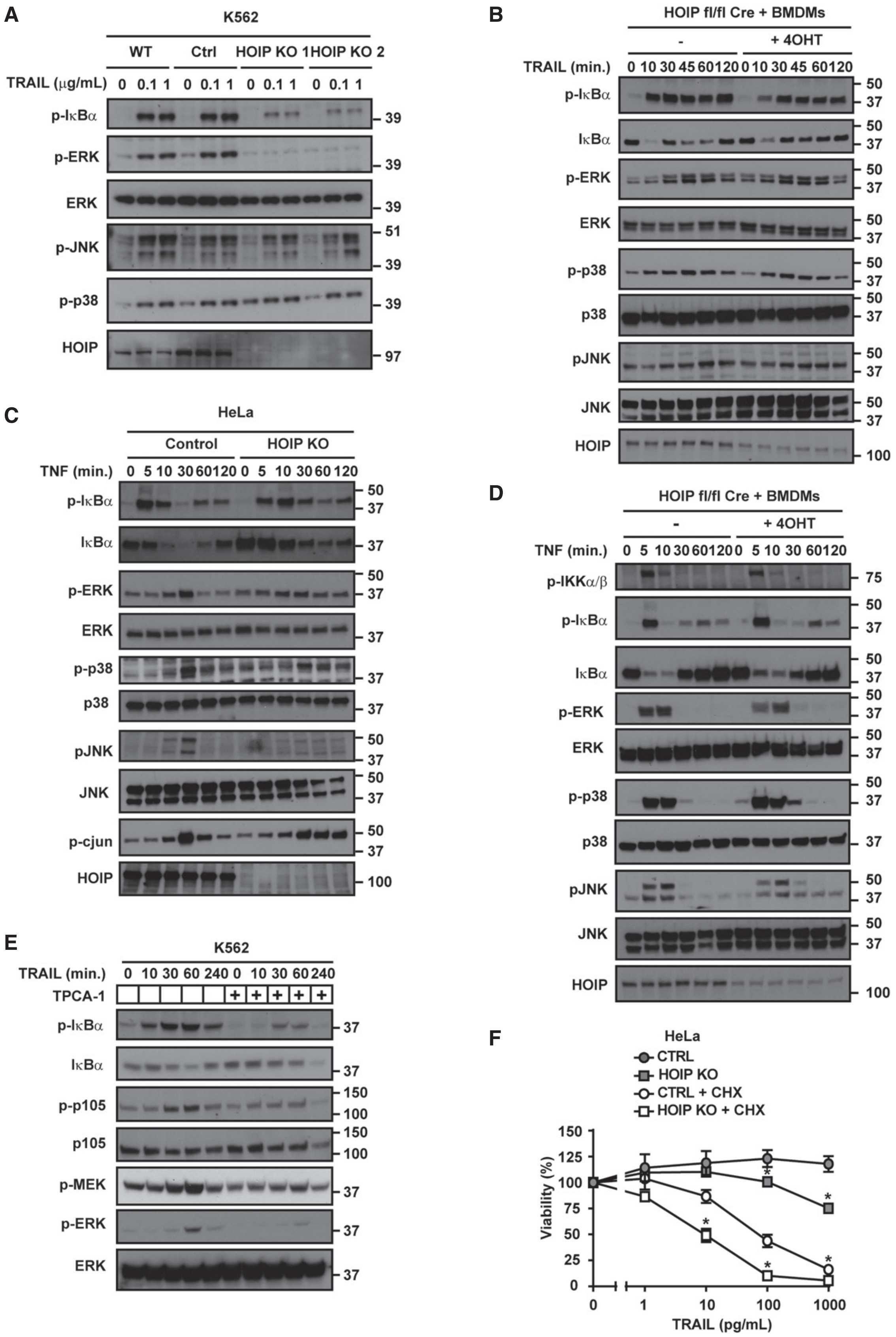

Figure EV3.

 © 2017 The Authors

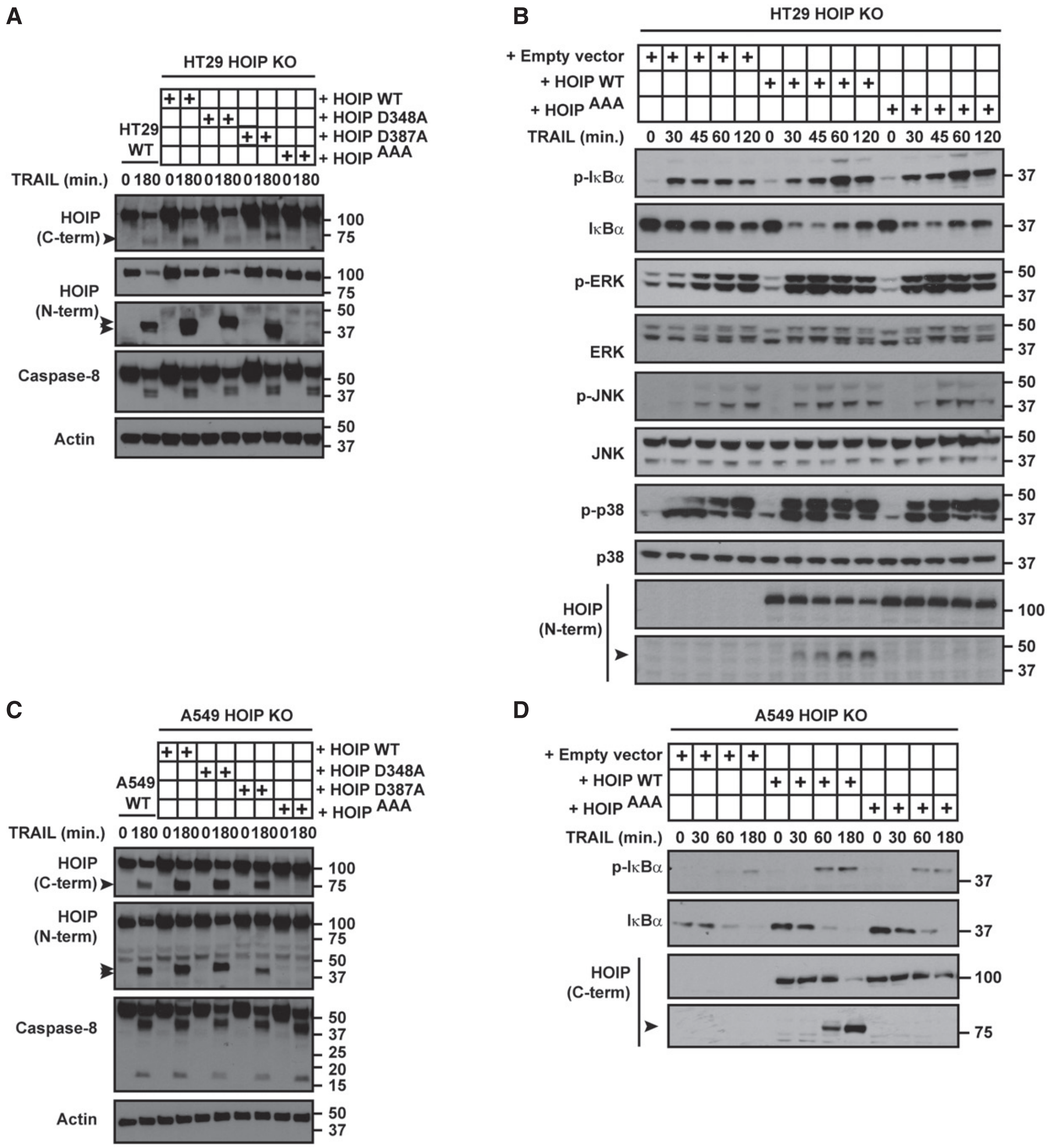

Figure EV4. TRAIL-induced caspase-dependent cleavage of HOIP does not alter its gene-activatory function (related to Fig 7).

A WT and HOIP KO HT29 reconstituted with HOIP WT, HOIP D348A, HOIP D387A or $HOIP^{AAA}$ were incubated with iz-TRAIL (1 μg/ml) for the indicated times and lysates were analysed by Western blot. Black arrowheads indicate the cleaved forms of HOIP.

B HOIP KO HT29 reconstituted with empty vector, HOIP WT or $HOIP^{AAA}$ were incubated with iz-TRAIL (500 ng/ml) for the indicated times and lysates were analysed by Western blot. The black arrowhead indicates the cleaved form of HOIP.

C WT and HOIP KO A549 reconstituted with HOIP WT, HOIP D348A, HOIP D387A or $HOIP^{AAA}$ were incubated with iz-TRAIL (1 μg/ml) for the indicated times and lysates were analysed by Western blot. Black arrowheads indicate the cleaved forms of HOIP.

D A549 HOIP KO reconstituted with HOIP WT or $HOIP^{AAA}$ were incubated with iz-TRAIL (200 ng/ml) for the indicated times, and lysates were analysed by Western blot. The black arrowhead indicates the cleaved form of HOIP.

Figure EV5. HOIP promotes IKK complex recruitment to TRAIL complex I and II and ensuing activation of NF-κB (related to Fig 8).

A Control and HOIP KO HeLa cells, pre-treated for 1 h with zVAD, were treated with FLAG-lz-TRAIL (500 ng/ml) for the indicated times. TRAIL complex I was immunoprecipitated via anti-FLAG beads, and complex II was immunoprecipitated from complex I-depleted lysates using anti-caspase-8-coupled beads. Western blot was performed using the indicated antibodies.

B Control and FADD KO A549 cells, pre-treated for 1 h with zVAD, were treated with FLAG-lz-TRAIL (500 ng/ml) for the indicated times. TRAIL complex I was immunoprecipitated via anti-FLAG beads, and samples were analysed by Western blot.

C LUBAC is recruited to the TRAIL-R-associated complex I downstream of FADD, caspase-8 and cIAP1/2, yet independently of RIPK1. LUBAC ubiquitinates several components, including caspase-8 and RIPK1, limiting the activation of caspase-8 and promoting the recruitment of the IKK complex to complex I and II. LUBAC thereby restricts apoptosis and promotes cytokine production. LUBAC also limits the recruitment of RIPK3 and MLKL to complex II, limiting necroptosis.

 © 2017 The Authors

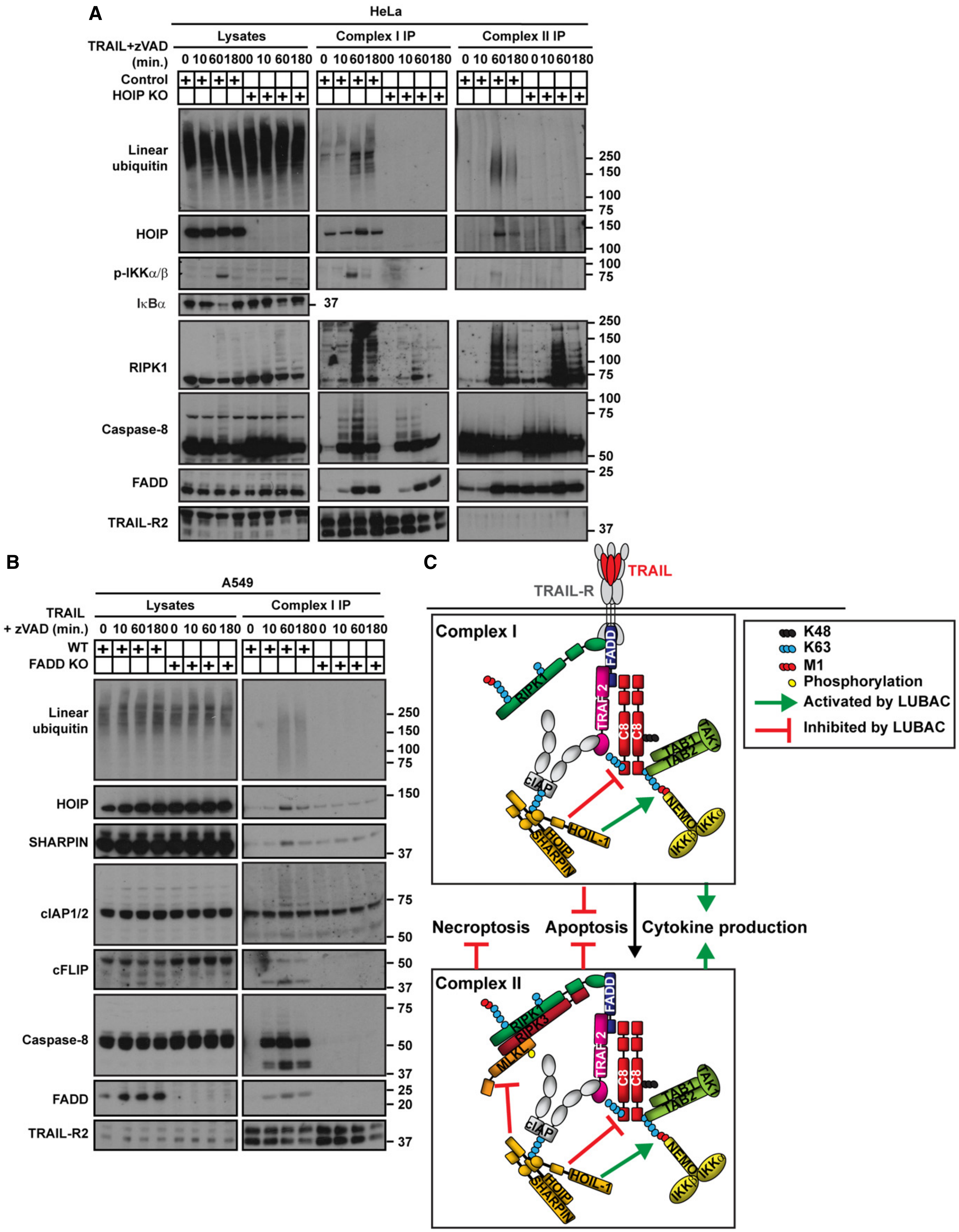
A
HeLa
TRAIL+zVAD (min.)
Lysates
Complex I IP
Complex II IP
Control
HOIP KO
Linear ubiquitin
HOIP
p-IKKα/β
IκBα
RIPK1
Caspase-8
FADD
TRAIL-R2
B
A549
TRAIL + zVAD (min.)
Lysates
Complex I IP
WT
FADD KO
Linear ubiquitin
HOIP
SHARPIN
cIAP1/2
cFLIP
Caspase-8
FADD
TRAIL-R2
C
TRAIL
TRAIL-R
Complex I
Complex II
K48
K63
M1
Phosphorylation
Activated by LUBAC
Inhibited by LUBAC
Necroptosis
Apoptosis
Cytokine production

Figure EV5.
